# Wildfire Smoke and Cardiorespiratory Emergency Visits in New Mexico 2022: Sensitivity to Exposure Estimates and Referent Periods

**DOI:** 10.1029/2025GH001492

**Published:** 2026-07-14

**Authors:** Olivia Sablan, Bonne Ford, Colin B. Hawkinson, Leiqiu Hu, Jihoon Jung, Chelsea Eastman Langer, Courtney Maichak, Kamal Jyoti Maji, Stephanie Moraga‐McHaley, Armistead G. Russell, Christopher K. Uejio, Melissa VanSickle, Emily V. Fischer, Jeffrey R. Pierce, Sheryl Magzamen

**Affiliations:** ^1^ Department of Atmospheric Science Colorado State University Fort Collins CO USA; ^2^ Cooperative Institute for Research in the Atmosphere Colorado State University Fort Collins CO USA; ^3^ New Mexico Department of Health Santa Fe NM USA; ^4^ Department of Atmospheric and Earth Science University of Alabama Huntsville Huntsville AL USA; ^5^ Department of Geography and the Environment University of North Texas Denton TX USA; ^6^ Department of Environmental and Radiological Health Sciences Colorado State University Fort Collins CO USA; ^7^ Department of Civil and Environmental Engineering Georgia Institute of Technology Atlanta GA USA; ^8^ Department of Geography Florida State University Fort Collins CO USA

**Keywords:** air quality, wildfire smoke, environmental health

## Abstract

In 2022, New Mexico (NM) experienced a number of wildfires, including the state's largest, Calf Canyon/Hermit's Peak. This study aimed to evaluate how different exposure estimate methods and referent period selection impacted associations between wildfire smoke and health outcomes using a case‐crossover study design. We investigated associations with exposure to fine particulate matter (PM_2.5_) from wildfire smoke and cardiorespiratory‐related emergency department (ED) visits in NM during 2022. Our study compared a range of exposure methods: (a) PM_2.5_ from the Environmental Protection Agency (EPA) regulatory‐grade monitors, (b) PM_2.5_ from both the EPA regulatory‐grade monitors and low‐cost PurpleAir observations, (c) modeled 24‐hr average wildfire smoke PM_2.5_ from the Community Multiscale Air Quality Modeling System (CMAQ), and (d) CMAQ daily 1‐hr maximum wildfire smoke PM_2.5_. The magnitude and statistical significance of health outcome associations varied substantially across exposure estimates and referent period selections. CMAQ‐based exposure estimates produced odds ratios with wider confidence intervals (CIs), while the product that leveraged both regulatory and bias‐corrected PurpleAir measurements improved the PM_2.5_ measurement spatial coverage and yielded epidemiological estimates with narrower CIs. This highlights the importance of low‐cost sensors in rural regions. Our findings emphasize the need to critically assess the inputs used in epidemiological studies for accurate and meaningful results, emphasizing the need for careful consideration of exposure assessment methods and study design when evaluating wildfire smoke health impacts.

## Plain Language Summary

It is important to understand how smoke affects human health in a warming climate. In 2022, New Mexico (NM) experienced the state's largest wildfire, Calf Canyon/Hermit's Peak. We used emergency department visits in NM to explore how different ways of measuring wildfire smoke exposure affected the results of a health study. We focused on fine particulate matter (PM_2.5_) exposure using four methods: (a) PM_2.5_ from the EPA regulatory‐grade monitors, (b) PM_2.5_ from both the EPA regulatory‐grade monitors and low‐cost PurpleAir observations, (c) modeled 24‐hr average wildfire smoke PM_2.5_ from the Community Multiscale Air Quality Modeling System (CMAQ), and (d) CMAQ daily 1‐hr maximum wildfire smoke PM_2.5_. Results varied depending on how smoke was estimated and the study design. The combination of regulatory monitors and low‐cost sensors improved the results because there was better coverage of monitors, highlighting the need for more air quality sensors. However, even with improved PM_2.5_ measurement coverage, the estimated health impacts were sensitive to methodological choices, such as how comparison days were selected. This study shows that decisions about how to measure smoke and health study design can strongly influence results, highlighting the need for careful consideration when evaluating the health impacts of wildfire smoke.

## Key Points

- Associations between wildfire smoke and cardiorespiratory emergency department visits varied by exposure product and referent period choice
- Combining regulatory‐monitors with corrected PurpleAir measurements improved spatial coverage, producing more stable epidemiological results
- In rural regions with sparse monitoring, exposure methods and case‐crossover design choices can influence wildfire smoke study conclusions

## 1 Introduction

Wildfires are increasing in duration, frequency, and intensity due to climate change (Abatzoglou et al., 2021; Westerling, 2016), leading to more widespread and increased wildfire smoke exposure across the United States (Burke et al., 2023; Childs et al., 2022; Volckens, 2024). The EPA National Emissions Inventory (NEI) consistently shows wildfires to be the largest source of primary PM_2.5_ in the US (US EPA, 2017, 2020), and exposure to smoke PM_2.5_ has been shown in previous studies to be associated with a suite of negative health outcomes. These outcomes include increased risk of cardiorespiratory morbidity (e.g., ED visits, hospitalizations, medication use, etc.) (Delfino et al., 2009; Gan et al., 2017; Hahn et al., 2021; Liu et al., 2015; Reid et al., 2016), particularly asthma‐related morbidity (Gan et al., 2020; Johnston et al., 2002; Rappold et al., 2011), adverse pregnancy outcomes (e.g., low‐birth weight) (Abdo et al., 2019; Holstius et al., 2012), and increased risk of mortality (Magzamen et al., 2021; O’Dell et al., 2021). With the increasing trend of wildfires expected to persist (Ford et al., 2018), we will likely see increasing rates of related morbidity and mortality in the future.

New Mexico (NM) suffered several large wildfires in 2022, including the largest in state history, the Calf Canyon/Hermit's Peak Fire. The Hermit's Peak fire started on 6 April 2022, and the Calf Canyon fire started on 19 April 2022. The two fires merged and were 100% contained by 21 August 2022, after burning a total of 1,382 km^2^ (Tunby et al., 2023). In total, NM experienced 748 wildfires total in 2022, burning about 3,700 km^2^ (National Interagency Fire Center, 2025). The Black Fire was the second largest and burned 1,316 km^2^ in southwestern NM May–June 2022 (Hulburd, 2022). Smaller wildfires also occurred in 2022, including the Cooks Peak Fire (InciWeb, 2024), the Cerro Pelado Fire (InciWeb, 2022b), and the Bear Trap Fire (InciWeb, 2022a).

Quantifying wildfire smoke exposure remains a major challenge, especially in regions like NM, where regulatory monitoring is sparse. While Environmental Protection Agency (EPA) monitors provide highly accurate and precise PM_2.5_ measurements, their limited coverage has led to growing interest in using low‐cost sensors. For example, PurpleAir low‐cost sensors have a large network that can be leveraged in the US; however, they are less accurate than regulatory monitors. Modeling wildfire smoke can provide data at high spatiotemporal resolution but may not always provide estimates that are representative of ground‐based monitoring. Accurately estimating smoke exposure is critical for epidemiological studies to produce meaningful results. In three studies conducted in Colorado, odds ratios (ORs), which express the likelihood of experiencing health impacts due to smoke exposure, associated with a 10 μg m^−3^ increase in wildfire smoke PM_2.5_ ranged from 0.9 to 2.2, with varying confidence intervals (CIs) (Alman et al., 2016; Magzamen et al., 2021; Stowell et al., 2019). This means that there were protective effects under some smoke‐impacted conditions (OR = 0.9), likely associated with behavior changes when residents were highly aware of the presence of local smoke. These studies used different smoke exposure estimates, which contributes to variations in results. Gan et al. (2017) also found that different smoke estimation methods resulted in varying associations with health outcomes. Through this study, we aim to evaluate how the choice of exposure estimate affects epidemiological results in a region with limited in situ monitoring.

The case‐crossover study design has been widely used to evaluate the health effects of smoke exposure (Gan et al., 2017, 2020; Hahn et al., 2021; Magzamen et al., 2021); however, the sensitivity of the selection of the referent period has not been explored. The case‐crossover design is valuable because it controls for individual‐level confounders (e.g., age, sex, race). Selection of an appropriate referent period, or the time period which the exposure event is compared to, is a critical component of the case‐crossover framework (Janes et al., 2005a, Janes et al., 2005b). In this analysis, we used a time‐stratified technique, which has been shown to address some challenges associated with referent periods (Janes et al., 2005a). The potential for different referent period choices to influence case‐crossover studies of short‐term pollution impacts is not well understood. This is especially relevant in NM during the Calf Canyon/Hermit's Peak fire, when some regions experienced nearly continuous smoke exposure for an extended period. Investigating the impact of different referent periods in a case‐crossover study provides insight to the sensitivity of the results.

We investigated the dependencies of inputs on ORs associated with wildfire smoke exposure in NM during summer 2022. We tested several smoke estimates, including in situ measurement‐based estimates and chemical transport model output. Additionally, we used two different referent periods to determine the impact on our results. By evaluating exposure estimates and referent period selection, we identify key uncertainties with assessing the impact of smoke on human health, specifically in a region with limited in situ PM_2.5_ monitoring during an intense wildfire season.

## 2 Methods

### 2.1 Study Area

**Figure 1:**
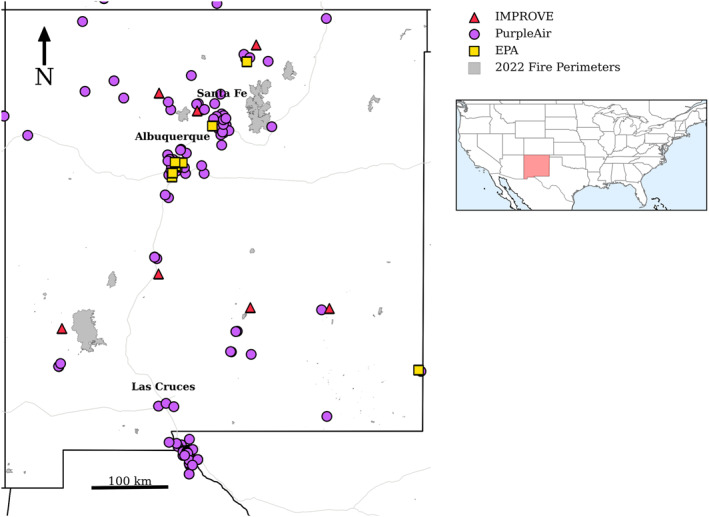
Map of study region and ground‐based PM_2.5_ sensors including the Environmental Protection Agency regulatory monitors (yellow squares), the Interagency Monitoring of PROtected Visual Environments monitors (red triangles), and the PurpleAir sensors (purple circles). The fire perimeters from the National Interagency Fire Center (2025) during 1 April–30 September 2022, are shaded gray. The map of the US is provided on the top right, with our study region (New Mexico) highlighted in red.

The study area includes all regions in the state of NM that have a ZIP code reported in the US Census Bureau 2010 5‐Digit ZIP Code Tabulation Area (ZCTA) (US Census Bureau, 2010). We did not use the 2020 ZCTA data; however, there were minimal changes from 2010, and our results would not be impacted. Due to a majority of NM being rural, there are several areas where there are no ZCTAs. In 2022, NM had 11 active EPA regulatory PM_2.5_ monitors, which are primarily located in more populated urban centers (e.g., Albuquerque, Santa Fe, Las Cruces). There were seven Interagency Monitoring of PROtected Visual Environments (IMPROVE) monitors in the area during 2022. IMPROVE monitor data are available through the EPA Air Quality System (AQS), but the primary purpose of the network is to monitor visibility trends in US National Parks and Wilderness Areas (Hand et al., 2024; Malm et al., 1994). IMPROVE monitors report measurements every 3 days, whereas monitors used for the National Ambient Air Quality Standards typically have hourly or daily observations. Additionally, NM had 91 low‐cost PurpleAir PM_2.5_ sensors at the time of the study (Figure 1). Despite integrating several data sources of PM_2.5_ observations, there is a lack of monitoring in the northeastern region of NM, where the largest wildfire in state history occurred in 2022.

#### 2.1.1 Study Population

NM has a population of ∼2.1 million people throughout 315,194 km^2^ of area (US Census Bureau, 2023). It is the fifth largest state by land mass but sparsely populated. The majority of the population identifies as Hispanic/Latino (50%) or American Indian and Alaska Native (11%). Nearly 18% of the NM population lives below the poverty line, where poverty is computed by the U.S. Census Bureau (2024) using income and family size in conjunction with defined Poverty Thresholds (US Census Bureau, 2023). Around 20% of the total population of NM is age 65 or older (US Census Bureau, 2023). The counties in northeastern NM, which were more impacted by the Calf Canyon/Hermit's Peak wildfire in 2022, have a higher population of people 65 or older in age. About 41% of Harding County (northeast of Las Vegas, NM), is 65 or older (US Census Bureau, 2023).

### 2.2 Smoke Exposure Estimates

We used four smoke PM_2.5_ estimates for our 2022 analysis: (a) PM_2.5_ from the EPA regulatory‐grade monitors, (b) PM_2.5_ from both the EPA regulatory‐grade monitors and low‐cost PurpleAir observations, (c) modeled 24‐hr average wildfire smoke PM_2.5_ from the Community Multiscale Air Quality Modeling System (CMAQ), and (d) CMAQ daily 1‐hr maximum wildfire smoke PM_2.5_. Conducting our analysis with four different exposure estimates for 2022 allowed us to assess the usefulness of each product in a case‐crossover study in NM.

#### 2.2.1 Regulatory‐Only Smoke Product

We used the 24‐hr smoke product from O’Dell et al. (2019) as our “Regulatory‐only” estimates. These estimates are produced by spatially interpolating in situ PM_2.5_ measurements from the EPA AQS. This includes EPA regulatory monitors (yellow squares in Figure 1) and IMPROVE monitors (red triangles in Figure 1) in NM and the surrounding states. To spatially interpolate, we used ordinary kriging, a geostatistical method that has been used in air quality research, to estimate PM_2.5_ concentrations between monitoring sites across a gridded surface (Isaaks & Srivastava, 1989; Janssen et al., 2008; Lassman et al., 2017). Altitude was not included in the kriging, which could influence smoke distribution in mountainous regions, though most regulatory monitors are located in populated areas outside of complex terrain. PM_2.5_ estimates are calculated for total (all‐source) PM_2.5_ and smoke PM_2.5_, where PM_2.5_ attributable to smoke is determined by the presence of HMS smoke plumes from the National Oceanic and Atmospheric Science Hazard Mapping System (NOAA HMS) (“Hazard Mapping System Smoke Product,” 2022). The HMS smoke plume product has several limitations. HMS does not distinguish fire type (i.e., prescribed fire, wildfire, agriculture fire); therefore, the smoke may not be exclusively from wildfires. We studied the wildfire season to decrease the likelihood of smoke from other types of fires. Additionally, smoke may not be observed by HMS during cloudy conditions, overnight periods, or when smoke is dilute (Sablan et al., 2026). HMS identifies smoke throughout the column thus smoke plumes aloft may be detected but may not impact the surface.

The Regulatory‐only product includes 24‐hr average PM_2.5_ on a 15 × 15 km grid for the contiguous US. Seasonal background concentrations for four climatological seasons (December‐February, March‐May, June‐August, September‐November) were determined by calculating the median PM_2.5_ at each grid cell on days without NOAA HMS smoke plumes and were removed from the PM_2.5_ smoke estimates.

We used the NASA Socioeconomic Data and Applications Center (SEDAC) world population (GPWv4) to calculate population‐weighted ZIP code‐level daily averages (Center for International Earth Science Information Network, 2023). We determined the fractional overlap of each grid with a ZCTA. Thus, a concentration is the sum of all gridded concentrations (Pop_*i*_) multiplied by the population (Pop_*i*_, regridded) (Center for International Earth Science Information Network, 2023) and the fractional overlap (*f*_*i*_) of the grid (*i*) and the ZIP code, divided by the total population in the ZIP code, as shown in Equation 1.

(1) $${\text{PM}}_{2.5,\text{ZIP}\;\text{Code}}=\frac{\Sigma_{i=1}^{n}f_{i}*{\text{Pop}}_{i}*{\text{PM}}_{i}}{\Sigma_{i=1}^{n}f_{i}*{\text{Pop}}_{i}}$$

#### 2.2.2 Regulatory + PA Smoke Product

Because the majority of the EPA AQS regulatory monitors are located in more populated areas, we incorporated low‐cost sensors (PurpleAir) to the O’Dell et al. (2019) (Regulatory‐only) product, allowing for greater in situ coverage of the largely rural state. We refer to this product as “Regulatory + PA.” PurpleAir sensors are low‐cost (∼$300 USD) and commercially available. The sensors contain two Plantowers (channels A and B), which use light‐scattering techniques to estimate PM_2.5_ mass at 680 ± 10 nm every 2‐min. PurpleAirs also have a BOSCH BME280 to measure pressure, temperature, and humidity. They have been tested in various settings (Barkjohn et al., 2021; Jaffe et al., 2023; Malings et al., 2020; Ouimette et al., 2022; Tryner et al., 2020) and have been shown to perform best for particles of size 0.3–1 μm (Kuula et al., 2020; Molina Rueda et al., 2023).

We took 10‐min averages of PurpleAir channels A and B for our data quality procedures. Data were removed from the analysis when: (a) channels A and B had an absolute difference of more than 10 μg m^−3^ or a relative difference of more than 10%, (b) the measurement was outside of the range of 0–1,000 μg m^−3^, (c) there was no corresponding humidity measurement, which was needed for the subsequent correction factor, (d) the A and B channels' correlation coefficient over the entire time period of available data was less than 0.75 (O’Dell et al., 2022). After completing our quality control procedures, we applied the Barkjohn et al. (2022) correction factor. This correction factor was developed by the US EPA for extreme wildfire smoke PM_2.5_. It reduces bias in PurpleAir estimates by scaling based on the measured concentrations and relative humidity. PurpleAir sensors exhibit higher uncertainty at elevated concentrations (Sayahi et al., 2019; Tryner et al., 2020); during our study period, 13 monitoring days experienced concentrations exceeding 100 μg m^−3^. While applying the Barkjohn correction factor reduces measurement bias, uncertainty in the PurpleAir estimates remains.

We spatially interpolated 24‐hr average PurpleAir and regulatory‐monitor PM_2.5_ measurements using ordinary kriging (described in Section 2.1.1) to a 0.1° × 0.1° grid of NM. By creating this product specifically for NM rather than at the national scale used by O'Dell et al. (2019) for the Regulatory‐only product, we were able to use a finer spatial resolution with the aim to better resolve local variability in smoke exposure across a large range of ZIP code sizes. We optimized the kriging parameters (i.e., sill, range, nugget) by leave‐one‐out cross‐validation (LOOCV) to maximize the correlation and minimize the error between the interpolated value and the measurement over the full year, as we used fixed variogram parameters for the entire study period. However, performance varied across days, and no single set of variogram parameters consistently performed well for all locations, likely due to spatial variability of wildfire smoke. When using the same variogram parameters as O’Dell et al. (2019) in our analyses, we had similar results to the optimized parameters (Figure S1 in Supporting Information S1), with the largest difference in ORs of 0.20. We used the optimized parameters in the following case‐crossover results. To evaluate the performance of the kriged PM_2.5_ estimates, we conducted a separate LOOCV analysis by iteratively removing monitors from the kriging analysis, which yielded an *R* ^2^ of 0.25 across all NM sites. This relatively low correlation likely reflects the sparsity of the monitoring network in NM. Additional comparisons of the kriged results are provided in Section 2.2.4.

To determine the presence of smoke we used the presence of HMS smoke plumes overlapping the grid cell, with the addition of a 25 km buffer around each plume to account for dilute smoke not identified by HMS. We selected this buffer after visually inspecting days when monitoring sites showed elevated PM_2.5_ concentrations slightly outside HMS plumes. We tested several buffer distances (i.e., no buffer, 10 km, 25 km) and determined the 25 km was best suited for this analysis because the distance between monitoring sites was large. The smaller tested buffers did not make a meaningful improvement to the smoke identification.

We removed the seasonal background PM_2.5_ concentration from our smoke estimates, similarly to O’Dell et al. (2019). Because smoke was present for most of spring and summer months in 2022, we used only two seasons (smoke season: April–August; non‐smoke season: January–March, September–December), in contrast to the four climatological seasons used in O’Dell et al. (2019). The 24‐hr average smoke PM_2.5_ concentration for each grid cell was determined by subtracting the background concentration from the total PM_2.5_ concentration on days with a HMS smoke plume over the grid. Negative values were set to zero.

#### 2.2.3 CMAQ 24‐hr Average and 1‐hr Maximum PM_2.5_

Maji et al. (2024) used the Community Multiscale Air Quality Modeling System (CMAQ, version 5.4) to model PM_2.5_ from six selected wildfires in NM during 6 April 2022–22 August 2022. These fires represented approximately 90% of the burned area during this period. However, burned area is not a direct measure of smoke emissions or PM_2.5_ contribution, as emissions depend on fuel type, combustion efficiency, and fire behavior, and smaller fires can contribute disproportionately to smoke under certain conditions. While the majority of smoke was likely produced by larger fires, smoke from small fires was not included in this model, which could lead to an underestimation of smoke PM_2.5_. Additionally, Maji et al. (2024) does not take into account the potential impacts of smoke from any other source not included in the emissions (e.g., prescribed fire smoke, smoke transported from other regions). We used estimates of 24‐hr mean smoke PM_2.5_ concentrations and the 1‐hr daily maximum smoke PM_2.5_ concentrations from this model, which were bias‐corrected for the season using routine monitoring network data. We included both the 24‐hr average and 1‐hr maximum to compare how each measure would impact the epidemiological results.

#### 2.2.4 Comparison of Total PM_2.5_ Estimates to In Situ Measurements

We compared total gridded PM_2.5_ to all ground‐based monitors (PurpleAir and EPA AQS sites) during 6 April–22 August 2022, for the four products (Figure S2 in Supporting Information S1). This comparison serves to evaluate the CMAQ model output and assess whether regulatory monitors alone capture the spatial variability observed by PurpleAir sensors. Each product was gridded at 15 × 15 km, and the monitor observations were compared to the overlapping grid cell. The Regulatory‐only product (*R* ^2^ = 0.13) and Regulatory + PA product (*R* ^2^ = 0.51) correlate more to all ground‐based monitoring sites than the CMAQ 24‐hr average (*R* ^2^ = 0.09) and CMAQ 1‐hr maximum (*R* ^2^ = 0.04). The Regulatory + PA product mean absolute difference (1.49 μg m^−3^) is lower than the other products (Regulatory only: 2.86 μg m^−3^; CMAQ 24‐hr average: 2.72 μg m^−3^; CMAQ 1‐hr maximum: 9.66 μg m^−3^). The fractional mean bias for the Regulatory + PA product (<0.01%) is also lower than the other products (Regulatory‐only: 0.17%; CMAQ 24‐hr average: 12%; CMAQ 1‐hr maximum mean bias: 1.48%). Lower values for the Regulatory + PA product are expected, since this product incorporates PurpleAir in situ measurements, where the other estimates do not. We could not directly compare our smoke PM_2.5_ estimates that were used in this paper to in situ measurements because we lack the ability to determine whether observed PM_2.5_ originated from smoke or other sources.

There is a relatively low R^2^ for the Regulatory‐only product compared to the Regulatory + PA product because we used all ground‐based monitors (PurpleAir and EPA AQS sites) for this evaluation, and the Regulatory‐only product does not use PurpleAir sensors to make estimates. The poor correlation between the Regulatory‐only product and the PurpleAir sensors emphasizes that the low‐cost sensors are monitoring more spatial variability than what is observed by the EPA AQS monitors. This suggests that the regulatory‐monitor density may be too low to capture wildfire smoke exposure. Additionally, we optimized the kriging parameters in the Regulatory + PA product for the spatial interpolation using the full year of observations, and the Regulatory‐only product uses several years to develop the kriging parameters. If we had used kriging parameters derived for each day, the correlations for both products would be higher; however, daily optimization increases computational demands and reduces repeatability, which limits its applicability for long‐term or large‐scale epidemiological studies. We instead chose fixed kriging parameters for the whole season consistency with prior work and to make the product more reproducible.

### 2.3 Measures of Health Outcomes

We retrieved 2022 ZIP code‐level daily counts of emergency department (ED) visits, through the NM Department of Health databases. Non‐federal facilities (i.e., Indian Health Services (IHS) and Veterans Affairs (VA) facilities) are not included in this data set, thus undercounting those populations. We investigated a suite of ED primary diagnoses, including visits for asthma morbidity, pneumonia, acute bronchitis, chronic obstructive pulmonary disease (COPD), heart failure, cardiac arrest, heart arrhythmia, ischemic heart disease, myocardial infarction, cerebrovascular disease, and cardiovascular disease. We also investigated “all respiratory” and “all cardiovascular” related ED visits, which include a broader range of billing codes. The ICD‐10 codes defining each diagnosis are provided in Supporting Information S1 (Table S1).

For this analysis, we used health data from patients who reported a billing address with a valid NM ZIP code. This excludes patients who are not NM residents and individuals who are unhoused (2% of total visits). There were also several records with erroneously reported ZIP codes (e.g., “XXXXX,” less than 5 digits, etc.) that were removed (1% of total visits).

#### 2.3.1 Health Data Quality Procedures

We removed duplicates from the ED data, when single individuals were recorded as having visited a facility two or more times on a given date using the patient identification number (<1% of total visits). All diagnosis codes from duplicate records were included in the singular record that was used for analysis when multiple records existed with different codes. Because patient identification numbers are facility‐specific, it was not possible to remove duplicate records if a patient was transferred to another facility. Although transferring is not very common with ED visits, this could result in potential overcounting of incident outcomes.

Patients may have visited the ED multiple times during the duration of our study. Due to the nature of our analysis, ED visits must be a rare event and not a frequent occurrence. We used the patient identification number to remove all facility visits following the first recorded visit during the study period (1% of total visits). Excluding subsequent visits may remove incident exacerbations from later smoke episodes, which could introduce bias. However, prior work suggests that for rare outcomes like ED visits, results are similar when using all events versus first events only (Gan et al., 2020). Since identification numbers are facility‐specific, we cannot remove multiple visits for patients who visited several different facilities in NM, again resulting in potential overcounting of incident outcomes.

### 2.4 Merging Health Records and Smoke Exposure Estimates

We used the US Census Bureau 2010 5‐Digit ZCTA shape files to produce population‐weighted smoke exposure estimates (US Census Bureau, 2010). In NM, there are several ZIP codes that correspond to Postal Office (PO) boxes, which do not contain geographic/spatial coordinates. ZIP codes relating to PO boxes were not included in the 2010 ZCTA files and were excluded from the subsequent analysis. Less than 2% of health data did not match to the ZCTA file.

### 2.5 Time‐Stratified Case‐Crossover Study Design

To evaluate the associations between health outcomes and PM_2.5_ from smoke, we used a time‐stratified case‐crossover study design (Gan et al., 2017, 2020; Hahn et al., 2021; Janes et al., 2005a, Janes et al., 2005b; Magzamen et al., 2021). In this method, the reported case is used as its own control, which adjusts for individual‐level confounders (e.g., age, sex, race). To account for exposures causing delayed health effects, we used a distributed lag‐model for days 0–5, where which lagged smoke PM_2.5_ exposures was included simultaneously and modeled using a natural spline with three degrees of freedom (Gasparrini et al., 2017). This approach allowed us to estimate lag‐specific effects for each day (lags 0–5), rather than averaging across lag days.

We matched 24‐hr (and 1‐hr maximum) smoke PM_2.5_ data to each corresponding case. We compared the smoke PM_2.5_ for each case to the same day of the week within the referent period. We evaluated two different referent periods: 1 April to 30 September 2022, and 6 April to 22 August 2022. The shorter referent period was selected because the CMAQ smoke data was only produced during the Calf Canyon/Hermit's Peak fire (6 April–22 August 2022) (Maji et al., 2024). By conducting this sensitivity analysis, we acknowledge the ZIP codes may have had constant smoke exposure in the shortened referent period, which limited the exposure contrast necessary for the case‐crossover design and contributed to the differences in results between the two referent periods. We used conditional logistic regression to estimate associations between cardiorespiratory ED visits and a 10 μg m^−3^ increase in PM_2.5_.

The daily population‐weighted heat index was used as a time‐varying confounder and lagged over days 0–5 with the daily smoke PM_2.5_. The heat index was calculated using 4 × 4 km daily PRISM data (https://prism.oregonstate.edu/), which was averaged to ZIP‐code level and population‐weighted using NASA Socioeconomic Data and Applications Center (SEDAC) gridded world population (GPWv4) (Center for International Earth Science Information Network, 2023). We compared several PM_2.5_ smoke products by conducting our analysis for each data set, using the ED data for 2022. We calculated ORs with 95% CIs.

## 3 Results

### 3.1 Wildfire Smoke PM2.5 Seasonal Overview

The PM_2.5_ ground‐based monitors in NM measured varying concentrations during 1 April–30 September 2022 (Table 1). Daily (24‐hr) PM_2.5_ concentrations ranged from 0–198 μg m^−3^ across all monitors (i.e., EPA regulatory monitors, IMPROVE monitors, PurpleAir sensors), with a standard deviation of 44.8 μg m^−3^. About 14% of days during the wildfire smoke season had at least one monitor in the state with an AQI classified as “Unhealthy for sensitive groups” (24‐ hour PM_2.5_ concentration ≥35.5 μg m^−3^ and ≤55.4 μg m^−3^) and 9% of the days had an AQI classified as “Unhealthy” (24‐ hour PM_2.5_ concentration ≥55.5 μg m^−3^ and ≤125.4 μg m^−3^) (U.S. Census Bureau, 2024) The maximum concentration of 198 μg m^−3^ was observed by a PurpleAir sensor on 24 June 2022 near the Calf Canyon/Hermit's Peak fire.

**Table 1:** State‐wide Average of New Mexico Wildfire Smoke and Total PM_2.5_ Average Concentrations (μg m^−3^) for the Four Population‐Weighted Smoke Estimates During Summer 2022

Exposure estimate	Dates in 2022	Smoke PM_2.5_ concentration (μg m^−3^)	Total PM_2.5_ concentration (μg m^−3^)
Regulatory‐only product	1 April–30 September	0.9 (2.4)	6.0 (3.9)
Regulatory‐only product	6 April–22 August	1.1 (2.7)	6.6 (4.2)
Regulatory + PA product	1 April–30 September	1.3 (2.8)	6.0 (3.9)
Regulatory + PA product	6 April–22 August	1.6 (3.1)	6.3 (4.2)
CMAQ 24‐hr average	6 April–22 August	0.3 (0.6)	5.7 (3.2)
CMAQ daily 1‐hr maximum	6 April–22 August	0.8 (2.1)	12.3 (9.7)

*Note.* Standard deviation between ZIP codes is reported in parentheses.

In 2022, the population‐weighted, state‐wide PM_2.5_ average for the Regulatory + PA smoke product was higher than the Regulatory‐only product during 1 April–30 September (1.3 vs. 0.9 μg m^−3^) as well as in the shorter period, during Calf Canyon/Hermit's Peak (1.6 vs. 1.1 μg m^−3^). The addition of PurpleAir sensors to the Regulatory‐only estimate provided more measurements of PM_2.5_ in northeastern NM, where Calf Canyon/Hermit's Peak occurred. There were more PurpleAir sensors in regions with higher percentages of days with HMS smoke plumes in the column during the wildfire (6 April–22 August 2022) than regulatory monitors (Figure S3 in Supporting Information S1); therefore, by only using the Regulatory‐only measurements, smoke is missed. The CMAQ 24‐hr average smoke PM_2.5_ is on average lower than the Regulatory‐only and Regulatory + PA product. The long‐term average of the CMAQ 1‐hr maximum is a more similar value to the Regulatory‐only and Regulatory + PA products. Substantial uncertainty remains in all exposure products.

**Figure 2:**
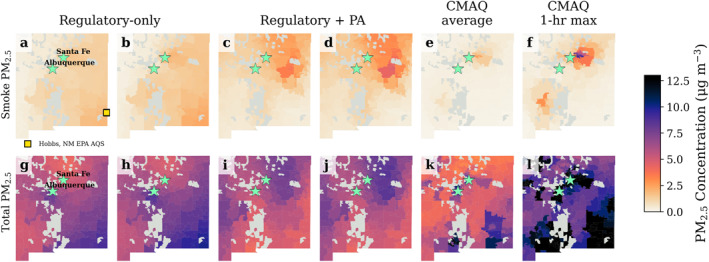
Comparison of ZIP code average population‐weighted smoke PM_2.5_ (top row) and total PM_2.5_ (bottom row) for 2022. The Regulatory‐only is shown for the entire wildfire season 1 April–September 30 (a and g) and for the duration of the Calf Canyon/Hermit's Peak wildfire (6 April–August 22; b and h). The Regulatory + PA product is also shown for the entire wildfire season (c and i) and the shortened period (d and j). The CMAQ 24‐hr average (e and k) and 1‐hr maximum (f and l) products are limited to 6 April–August 22. One regulatory monitor is shown with a yellow square in subplot a). Gray areas are not affiliated with ZIP codes. Two major cities are identified with cyan stars.

To investigate spatial variability in the smoke products, we compared estimates by ZIP code during summer 2022 (Figure 2). During the Calf Canyon/Hermit's Peak fire (6 April–22 August 2022), the ZIP code with the highest average smoke PM_2.5_ concentration in the Regulatory‐only product was 2.4 μg m^−3^ This occurred in Jal, NM (ZIP code: 88252) and was driven by a single nearby regulatory monitor in Hobbes, NM. All other regulatory monitors in NM were to the west of the Calf Canyon/Hermit's Peak wildfire and likely upwind. Although eastern NM experienced higher smoke PM_2.5_ concentrations than western NM, this product does not show a clear increase in smoke PM_2.5_ near the Calf Canyon/Hermit's Peak wildfire, likely due to the limited monitor coverage in close proximity to the fire. In comparison, the ZIP code with the highest average smoke PM_2.5_ concentration for the Regulatory + PA product occurred near the wildfire. Cuevro, NM (ZIP code: 88417), located slightly southeast of the fire, had the highest average PM_2.5_ of 4.6 μg m^−3^. The PurpleAir sensors located east of the regulatory monitors, closer to the fire, provided additional spatial coverage closer to the fire that contributed to higher smoke PM_2.5_ concentrations. The addition of PurpleAir sensors expanded spatial coverage near the wildfire, leading to different estimated exposure compared to the Regulatory‐only product, highlighting the importance of low‐cost monitors in regions where regulatory networks are sparse.

For both CMAQ products (24‐hr average and 1‐hr maximum), the highest average smoke PM_2.5_ concentration occurred at the wildfire location (ZIP code: 87731). Since Calf Canyon/Hermit's Peak was the largest wildfire included in the model, this result is expected. For the 24‐hr average product, the highest average smoke PM_2.5_ concentration was 2.6 μg m^−3^, and the highest concentration for the 1‐hr maximum product was 9.2 μg m^−3^. The highest CMAQ 24‐hr average smoke PM_2.5_ during the wildfire gave closer estimates to the Regulatory‐only and Regulatory + PA products. However, the CMAQ 1‐hr maximum had a wider spread of smoke impacts than the CMAQ 24‐hr product, with more ZIP codes with higher concentrations. The measurement‐based products showed a more similar pattern, with many smoke‐impacted ZIP codes near the wildfire. We conducted our subsequent epidemiological analysis with both the CMAQ 24‐hr average and the CMAQ 1‐hr maximum.

Estimates of total PM_2.5_ have diverse spatial patterns across the products. The Calf Canyon/Hermit's Peak fire is most distinguishable in total PM_2.5_ from the Regulatory + PA smoke product, with the highest ZIP code smoke PM_2.5_ estimate occurring near the fire (ZIP code: 88421: Garita, NM). For both CMAQ total PM_2.5_ estimates, there were also increased concentrations in southeast NM compared to central and northern NM. Because the model uses anthropogenic emissions from the EPA NEI, this increase in southeastern NM likely occurred due to oil and natural gas production in this region.

### 3.2 Descriptive Overview of Cardiorespiratory Emergency Department Visits

Cardiorespiratory‐related emergency department (ED) visits for all patients in NM with valid ZIP codes during 1 April–30 September 2022, and the corresponding age‐related, gender‐related, and race‐related strata are reported in Table 2. Data for the shortened referent period (6 April–22 August 2022) can be found in Supporting Information S1 (Table S2). During 1 April–30 September 2022, there were a total of 73,766 visits to the ED for the health outcomes of interest. However, because broader categories (e.g., all‐respiratory) encompass specific conditions (e.g., asthma), this total includes duplicate counts of visits classified under multiple health outcome categories. About 40% of the ED visits were due to all respiratory‐related outcomes (*n* = 29,489), compared to 20% for cardiovascular‐related outcomes (*n* = 15,478). Pneumonia accounted for the highest number of visits among individual health outcomes, representing 7% of the total.

**Table 2:** Summary of Cardiorespiratory‐Related Emergency Department (ED) Visits in New Mexico Between April 1–30 September 2022

		Age category			Sex		Race/Ethnicity					
Health outcomes	Cases (n)	<15 (%)	15 to 64 (%)	≥ 65 (%)	Male (%)	Female (%)	AIAN (%)	ANHPI (%)	Black (%)	Hispanic (%)	White (%)	Unknown (%)
All respiratory	29,489	30.7	49.7	19.6	46.7	53.3	9.0	0.7	2.7	49.4	33.9	4.3
Asthma	3,373	32.2	59.7	8.1	45.4	54.6	8.1	0.8	4.7	54.3	27.9	4.2
COPD	1,887	0.2	38.0	61.8	44.7	55.3	1.5	0.5	2.4	30.0	62	3.7
Pneumonia	5,102	15.1	42.9	42.1	50.2	49.8	6.4	0.5	2.4	42	45.4	3.3
Acute bronchitis	4,082	44.9	42.6	12.6	47.7	52.3	7.9	0.5	2.3	55.3	30.3	3.7
All Cardiovascular	15,478	0.3	43.5	56.2	53.9	46.1	3.7	0.8	2.6	36.7	51.6	4.4
Cardiac Arrest	487	4.1	46.2	48.9	61.2	38.8	5.1	0.8	4.3	38.6	39	12.1
Arrhythmia	3,121	0.2	38.5	61.3	52.6	47.4	2.8	0.8	1.4	26	63.5	5.4
Heart Failure	856	–	41	59	56.1	43.9	3.4	0.7	1.5	37.1	53.4	3.9
Ischemic Heart Disease	3,587	–	46.1	53.9	63.5	36.5	2.9	0.9	2.0	39.8	50.8	3.6
Myocardial Infarction	3,173	–	46.2	53.8	64.3	35.6	2.7	1.0	2.1	40.0	50.4	3.8
Cerebrovascular Disease	3,131	0.3	34.9	64.8	51.1	48.9	5.2	0.9	2.2	37.0	50.9	3.7
**TOTAL**	**73,766**	**9,097**	**21,540**	**14,850**	**22,349**	**23,137**	**4,678**	**537**	**1,923**	**32,225**	**31,308**	**3,095**

*Note.* American Indian/Alaskan Native is abbreviated as AIAN, and Asian/Native Hawaiian/Pacific Islander is abbreviated as ANHPI. Corresponding ICD‐10 codes are provided in Table S1 in Supporting Information S1 and a summary for ED visits from 6 April–22 August 2022, in Table S2 in Supporting Information S1.

NM has a higher proportion of people who identify as Hispanic/Latino and American Indian and Alaska Native compared to other US states, as shown in the demographic summary (Table 2). The largest percentage of people who visited the ED during this period were Hispanic (46%). The next largest was white (42%). There were over 12% more Hispanic patients who visited the ED for a respiratory‐related health outcome (49.4%) than Hispanic patients who visited for a cardiovascular‐related health outcome (36.7%); however, there were >17% more white patients with cardiovascular‐related health outcomes (51.6%) than respiratory‐related (33.9%). Our study did not include data from the IHS or VA facilities; therefore, our race‐related strata may not be completely representative of the entire NM population. About 6% of the NM population are veterans and 11% identify as American Indian/Alaskan Native (US Census Bureau, 2023), but the precise number of these populations who used IHS or VA facilities in 2022 is unknown.

### 3.3 Comparison of Smoke Estimates in Epidemiological Results

**Figure 3:**
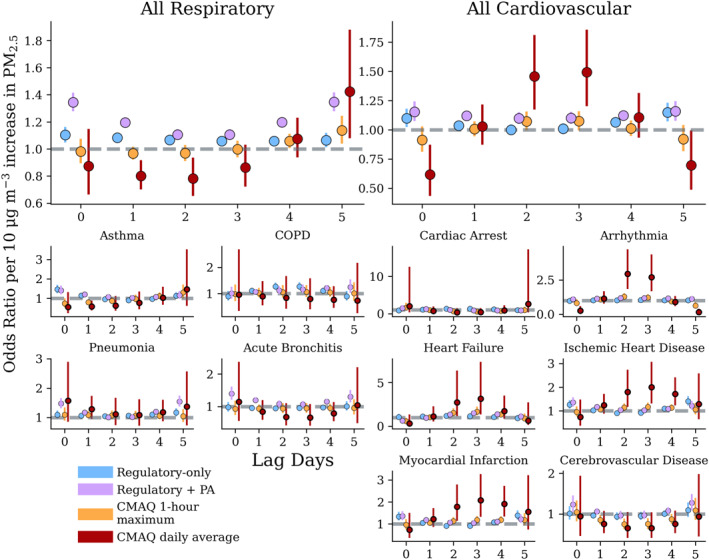
Distributed‐lag effects of a 10 μg m^−3^ increase in wildfire smoke PM_2.5_ on likelihood of cardiorespiratory emergency department visits during 6 April–22 August 2022 for the smoke products: Regulatory‐only (blue), Regulatory + PA (purple) and the CMAQ 1‐hr maximum (orange) and CMAQ 24‐hr average (red).

ORs from distributed‐lag models for a 10 μg m^−3^ increase in smoke PM_2.5_ exposure are shown in Figure 3 for the four smoke estimates: Regulatory‐only, Regulatory + PA, CMAQ 24‐hr average, and CMAQ 1‐hr maximum. To facilitate this comparison, we used the referent period 6 April–22 August 2022 because the CMAQ PM_2.5_ was only produced during this time. By using this shortened referent period, we acknowledge that some ZIP codes may have had constant smoke exposure, potentially affecting result accuracy. A more detailed comparison of referent period selection is provided in Section 3.4.

Associations with smoke exposure and cardiorespiratory ED visits varied by smoke product. For example, smoke PM_2.5_ exposure had a negative association for all respiratory‐related ED visits using the CMAQ 24‐hr average product (Lag 1 OR = 0.80, 95% CI = 0.70, 0.92; Lag 2 OR = 0.78, 95% CI = 0.65, 0.94); however, all other products show increased odds or results that were not significant. The ORs for the CMAQ 24‐hr average smoke PM_2.5_ estimates are much higher in magnitude, with larger CIs than the other products, suggesting this exposure estimate was not suitable for this epidemiological application. The largest OR for the CMAQ 24‐hr average is 3.16 (Heart Failure: Lag 3 OR = 3.16, 95% CI = 1.36, 7.36), compared to the largest OR for the Regulatory + PA product of 1.55 (Pneumonia: Lag 5 OR = 1.55, 95% CI = 1.38, 1.74). The largest CIs for the CMAQ 24‐hr average (Cardiac Arrest: Lag 5 OR = 2.58, 95% CI = 0.39, 17.18) is over 15 times larger than the largest interval for the Regulatory + PA product (Cardiac Arrest: Lag 0 OR = 1.50, 95% CI = 0.97, 2.31). Our epidemiological results change considerably across smoke exposure products.

Modeling smoke in this region is particularly challenging for prognostic models like CMAQ due to complex terrain and the dynamic nature of the local Calf Canyon/Hermit's Peak fire. These factors can increase the potential for exposure misclassification. Additionally, because the CMAQ estimates included only six fires in NM during 2022, they may underestimate smoke exposure. Smaller fires in NM occurred during this time period that were not included in the model, and there may have been transported smoke from other states, leading to an underestimation of smoke PM_2.5_ concentrations. All smoke estimates used in this study rely on the regulatory monitoring network, which may not provide sufficient spatial coverage to create quantitative smoke PM_2.5_ estimates in NM, particularly in rural and fire‐impacted regions. Expanding low‐cost sensor networks, such as PurpleAir, could improve exposure estimates in areas with limited monitoring. Given these limitations, the use of smoke PM_2.5_ estimates in epidemiological studies should be carefully evaluated on a case‐by‐case basis.

### 3.4 Epidemiological Results When Implementing Different Referent Periods

**Figure 4:**
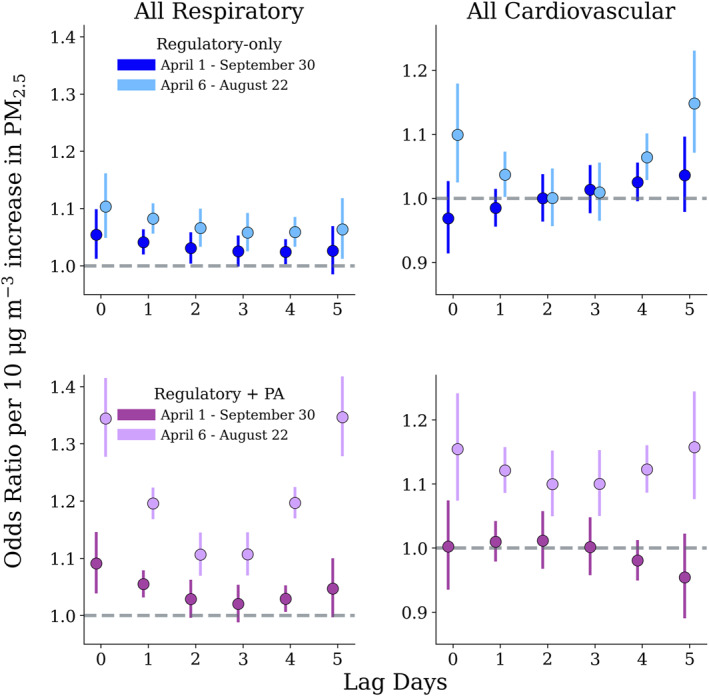
Distributed‐lag effects of a 10 μg m^−3^ increase in wildfire smoke PM_2.5_ on likelihood of all respiratory (left) and cardiovascular (right) related emergency department visits during 1 April–30 September 2022 and 6 April–22 August 2022 for two smoke products: Regulatory‐only (top; blue) top) and Regulatory + PA (bottom; purple).

We compared results of the distributed‐lag models for the Regulatory‐only and Regulatory + PA smoke products for two referent periods: 1 April–30 September 2022, and 6 April–22 August 2022 (Figure 4). Results are shown for all‐respiratory and all‐cardiovascular ED visits, with referent period comparisons for all individual ICD‐10 codes available in Supporting Information S1 (Table S1 and S2 in Supporting Information S1). The addition of PurpleAir observations to the EPA AQS monitors provided more PM_2.5_ measurements in regions with higher percentages of days with smoke in the atmospheric column (Figure S3 in Supporting Information S1), leading to an increase in smoke PM_2.5_ concentrations. While we acknowledge these differences, our focus in this section is to evaluate the impact of referent period selection.

The results across referent periods for all respiratory‐related ED visits with the Regulatory‐only product are more consistent compared to all‐cardiovascular as well as the all‐respiratory related results for the Regulatory + PA product. There were positive associations observed on Lags 0–2 and 4 for the 1 April–September 30 referent period with the Regulatory‐only product and for the 6 April–August 22 referent period (Figure 4). In comparison, all lagged days for the Regulatory‐only product had positive associations with all‐respiratory related ED visits for the shorter referent period during the wildfire. There were several lagged days for the Regulatory‐only product and the longer referent period without clear associations. There were no significant associations for the Regulatory‐only product and all‐cardiovascular related ED visits for the longer referent period. The shorter referent period for this product had positive associations for Lags 0–1 and 4–5. With Calf Canyon/Hermit's Peak burning for the duration of the 6 April–August 22 referent period, there were limited days with lower smoke PM_2.5_ concentrations to provide a meaningful comparison for exposure. While the shorter referent period showed more statistically significant associations, this likely reflects limited contrast in smoke PM_2.5_ during the wildfire. The longer referent period includes a smoke‐free period at the end of the season, providing better exposure contrast. However, the substantial differences in results between the two referent periods demonstrate the sensitivity of our findings to this design choice.

Similar to the Regulatory‐only smoke estimate, there were positive associations with all respiratory‐related ED visits and a 10 μg m^−3^ increase in smoke PM_2.5_ for both referent periods using the Regulatory + PA estimates. The magnitude of the ORs for the shorter referent period were higher than odds for the longer referent period. There were no clear associations for all‐cardiovascular related ED visits and the longer referent period; however all lagged days for the shorter referent period had positive associations. These findings emphasize that effect estimates are sensitive to referent period selection, particularly when the referent period is restricted to a prolonged smoke event.

### 3.5 Association With Smoke Exposure by Health Outcome

**Figure 5:**
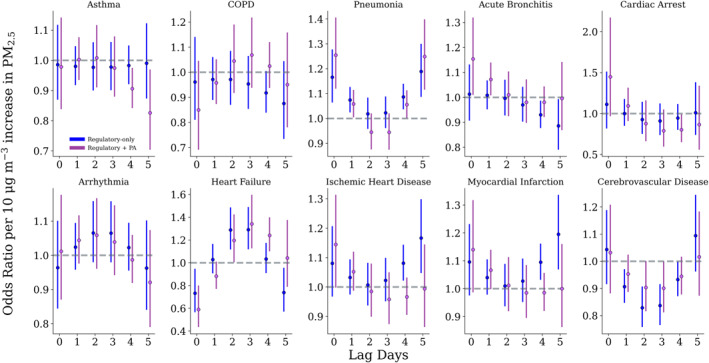
Distributed‐lag effects of a 10 μg m^−3^ increase in wildfire smoke PM_2.5_ on likelihood of cardiorespiratory emergency department visits during 1 April–30 September 2022 for the smoke products: Regulatory‐only (blue) and Regulatory + PA (purple).

In the following section, we present quantitative findings on association with a 10 μg m^−3^ increase in smoke PM_2.5_ and ED visits. We have shown that the selection of the referent period and the smoke estimation method can influence these associations; therefore, the following discussion should provide insight into overall patterns during the 2022 NM wildfire season but should not be interpreted as precise and absolute results. Additionally, we did not adjust for multiple comparisons across the various health outcomes and lagged days tested, meaning some significant associations may represent chance findings rather than true effects. However, our primary goal was to demonstrate how exposure product and study design choices influence epidemiological findings rather than to establish definitive health effects. We compare the Regulatory‐only product and the Regulatory + PA for the referent period 1 April–30 September 2022, as we have more confidence in these estimates and referent period (Figure 5).

Asthma morbidity is often associated with exposure to wildfire smoke (Gan et al., 2020; Johnston et al., 2002; Rappold et al., 2011; Reid et al., 2016); however, we did not observe this in our results. With the Regulatory + PA product, we observed protective effects for asthma on lag days 4–5 (Lag 4 OR = 0.91, 95% CI: 0.84, 0.98; Lag 5 OR = 0.83, 95% CI: 0.70, 0.97). There were no significant associations with the Regulatory‐only product. These results may be due to variations in smoke exposure estimates or misclassification of exposure, as we have demonstrated how different smoke estimates can influence results. There were also no significant associations observed for COPD, cardiac arrest, and heart arrhythmia for either product.

Other contextual factors may also explain the lack of positive associations between smoke exposure and asthma. Asthma is more prevalent in children than older adults (Global Asthma Network, 2022), and the most smoke‐impacted regions in NM had a large population of people 65 or older (US Census Bureau, 2023). This demographic difference may explain why our study did not find significant positive associations with asthma, as observed in previous studies. Additionally, there were evacuation orders in northeastern NM during the wildfire season (Baca & Schear, 2022), and health agencies strongly communicated about smoke risks. The communication with the public may have prompted behavior changes (e.g., sheltering indoors, using air filtration, etc.), which could have reduced exposure and asthma morbidity. These protective actions, combined with population characteristics, may have decreased the associations between wildfire smoke exposure and asthma‐related ED visits.

We observed significant increased risk in ED visits for pneumonia during Lags 0–1 for both the Regulatory‐only smoke product (Lag 0 OR = 1.17, 95% CI: 1.06, 1.28; Lag 1 OR = 1.07, 95% CI: 1.02, 1.13) and the Regulatory + PA smoke product (Lag 0 OR = 1.25, 95% CI: 1.12, 1.41; Lag 1 OR = 1.06, 95% CI: 1.00, 1.11). Pneumonia is caused by a suite of infectious respiratory pathogens with the highest prevalence in winter. The positive associations observed in this analysis with exposure to wildfire smoke may be a result of exposure misclassification. There were significant associations with acute bronchitis for the Regulatory + PA product, with the most inconsistent results between the Regulatory + PA and the Regulatory‐only product ORs on Lag 0 (difference: 0.14). These findings suggest a potential association between wildfire smoke exposure and respiratory infections, though differences between smoke exposure estimates highlight the need for careful consideration of exposure misclassification in epidemiological studies.

Our distributed‐lag model showed increased odds of heart failure on lag days 2 and 3 for the Regulatory‐only smoke product and Regulatory + PA. Older populations are often more susceptible to experiencing heart failure, and there was a large percentage of people 65 or older near the wildfire. Although heart failure has a strong significant association with smoke exposure in this analysis, when considering all cardiovascular‐related health outcomes (Figure 4), there is not a significant association. Previous studies have shown that exposure to wildfire smoke corresponds more consistently to respiratory‐related health effects than cardiovascular‐related (Liu et al., 2015; Reid et al., 2016). The increased odds for heart failure in this study may be influenced by the high proportion of residents age 65 and older in the smoke‐impacted region or potential misclassification of smoke exposure.

Overall, the Regulatory‐only and the Regulatory + PA product provide similar results for our distributed‐lag model for all health outcomes. The largest difference in ORs for these products occurred with cardiac arrest (Regulatory‐only: Lag 0 OR = 1.11, 95% CI: 0.82, 1.51; Regulatory + PA: Lag 0 OR = 1.45, 95% CI: 0.97, 2.17). For some health outcomes, the choice of smoke product impacted the significance and overall conclusions. For example, there are significant protective effects with the Regulatory‐only smoke estimates and cerebrovascular disease. This relationship was only present with the Regulatory + PA product on Lag 3 (OR = 0.90, 95% CI: 0.81, 0.99), with all other days having no significant associations. This again demonstrates the importance of selecting a smoke exposure estimate in epidemiological studies.

## 4 Conclusions

We investigated how different smoke exposure inputs to an epidemiological study impacted the assessment of health outcomes associated with wildfire smoke exposure in NM during 2022. The Calf Canyon/Hermit's Peak Fire occurred in northeastern NM April–August 2022. We used ED visits by ZIP code and a 10 μg m^−3^ increase in smoke PM_2.5_ to calculate the distributed‐lag effects. We compared four exposure estimates in our analysis: (a) PM_2.5_ from the EPA AQS monitors, (b) PM_2.5_ from both the EPA AQS monitors and low‐cost PurpleAir observations, (c) modeled 24‐hr average wildfire smoke PM_2.5_ from the Community Multiscale Air Quality Modeling System (CMAQ), and (d) CMAQ daily 1‐hr maximum wildfire smoke PM_2.5_. Modeling smoke PM_2.5_ for a dynamic fire in an environment with varying topography brings many challenges, and we determined the chemical transport model estimates (CMAQ) were not as useful for estimating exposure‐response relationships for acute events.

Low‐cost monitors were essential for increasing spatial resolution of PM_2.5_ monitoring, particularly in rural areas where regulatory monitors are sparse. In NM, most regulatory monitors are located in populated areas like Albuquerque, Santa Fe and Las Cruces, making them less effective for capturing wildfire smoke exposure in rural regions. Adding low‐cost sensors improved spatial coverage for our smoke exposure estimates, but large areas of the state likely impacted by smoke still lacked monitors. Therefore, gaps in monitors remained in our smoke estimates that used EPA AQS monitors and PurpleAir sensors, potentially leading to exposure misclassification. For ground‐based smoke estimates to be accurate, the monitoring network must be dense enough to capture spatial gradients in smoke exposure. This challenge is particularly evident with large, dynamic fires like Calf Canyon/Hermit's Peak, where smoke conditions changed rapidly across the landscape.

Selection of the referent period in our study impacted the results. We compared all‐cardiovascular and all‐respiratory related ED visits for two referent periods: 6 April–22 August 2022 (during Calf Canyon/Hermit's Peak) and 1 April–30 September 2022 (the entire wildfire season). Because the CMAQ output was only available for the shortened referent period, we conducted this sensitivity analysis to investigate exposure and adverse health outcome differences. The shorter referent period lacked contrast in smoke PM_2.5_ due to the large, prolonged wildfire. We found our results were inconsistent using different referent periods, demonstrating the sensitivity of this study design choice. Determining the most appropriate referent period remains a challenge, particularly as wildfire seasons extend in a warming climate.

We used the smoke estimates from the EPA AQS monitors and PurpleAir sensors to explore how exposure product selection affected associations between smoke exposure and individual health outcomes. The sensitivity of results to methodological choices was evident across health outcomes. We did not find a significant association with asthma, which has been found in other studies. Heart failure showed the strongest association compared to the other health outcomes, but results still varied by smoke estimate. These variable findings demonstrate that in sparse monitoring environments, exposure errors can substantially influence epidemiological conclusions, even when using the most spatially comprehensive product available (i.e., EPA AQS and PurpleAir combined).

There were many factors in 2022 that may have impacted our results. First, the COVID‐19 virus may have resulted in residents not visiting the ED for smoke‐related health concerns despite facing symptoms. In addition, many of the most smoke‐impacted areas were rural, where residents often live farther from care facilities, potentially deterring ED visits due to longer travel times. Mandatory fire evacuations in many communities may have reduced the number of affected residents or led them to seek healthcare in other regions. There may have also been behavioral changes (e.g., staying indoors, wearing masks outdoors, changing home filters, etc.), due to the strong communication from health agencies as well as the persistent visibility of smoke in the air, which may have led to a decrease in ED visits. In addition to wildfire smoke, NM is impacted by prescribed and agricultural fire smoke, as well as transported smoke from fires in other regions. In 2017, the largest source of landscape‐fire smoke PM_2.5_ emissions was wildfires. Emissions from prescribed and agricultural fires are much smaller than those from wildfires (US EPA, 2017), but still contribute to the PM_2.5_ concentrations. Smoke was transported to NM in 2022 from other regions, such as California, Arizona, and Mexico. PM_2.5_ composition varies between fires and with smoke age (Bian et al., 2020; Hodshire et al., 2019), which can impact health differently (Magzamen et al., 2021; Riss et al., 2025) and was not accounted for in this study. The varying sources of smoke have the potential to impact smoke PM_2.5_ exposure estimates for the products based on ground‐based measurements, while chemical transport model output only included wildfire smoke as a source.

The exposure inputs and study design choices in epidemiological studies are critical. Our analysis demonstrates that using different smoke exposure estimates and referent periods can lead to varying health outcome associations. This work highlights the difficulty in quantifying acute health‐effect estimates in a region with sparse surface PM_2.5_ monitoring. To improve smoke estimates, air quality monitoring networks must be expanded, especially in rural regions. As wildfires continue to grow in intensity and frequency due to climate change, developing improved exposure estimates and careful consideration of study design choices will be critical for informing public health responses and healthcare planning.

## Conflict of Interest

The authors declare no conflicts of interest relevant to this study.

## Supporting information

Supporting Information S1

## Data Availability

Population‐weighted smoke PM_2.5_ estimates for the four products (PM_2.5_ from the EPA AQS monitors; PM_2.5_ from both the EPA AQS monitors and low‐cost PurpleAir observations; modeled 24‐hr average wildfire smoke PM_2.5_ from the Community Multiscale Air Quality Modeling System (CMAQ); CMAQ daily 1‐hr maximum wildfire smoke PM_2.5_) by ZIP code and population‐weighted heat index by ZIP code can be accessed at (Sablan, 2025). The code written to conduct this analysis can be found here: https://github.com/oliviasablan/New_Mexico_Wildfire. The ED data in this manuscript include protected health information (e.g., patient identification number, patient ZIP code, etc.) covered by the Health Information Portability and Accountability Act. Therefore, these data are not available due to data use agreements with the NM Department of Health. Parties interested in reproducing or extending this work will need to set up their own data use agreements with the NM Department of Health to receive this data.

## References

[gh270179-bib-0001] Abatzoglou, J. T. , Battisti, D. S. , Williams, A. P. , Hansen, W. D. , Harvey, B. J. , & Kolden, C. A. (2021). Projected increases in Western US forest fire despite growing fuel constraints. Communications Earth and Environment, 2(1), 1–8. 10.1038/s43247-021-00299-0

[gh270179-bib-0002] Abdo, M. , Ward, I. , O’Dell, K. , Ford, B. , Pierce, J. R. , Fischer, E. V. , & Crooks, J. L. (2019). Impact of wildfire smoke on adverse pregnancy outcomes in Colorado, 2007–2015. International Journal of Environmental Research and Public Health, 16(19), 3720. 10.3390/ijerph16193720 31581673 PMC6801422

[gh270179-bib-0003] Alman, B. L. , Pfister, G. , Hao, H. , Stowell, J. , Hu, X. , Liu, Y. , & Strickland, M. J. (2016). The association of wildfire smoke with respiratory and cardiovascular emergency department visits in Colorado in 2012: A case crossover study. Environmental Health, 15(1), 64. 10.1186/s12940-016-0146-8 27259511 PMC4893210

[gh270179-bib-0004] Baca, L. , & Schear, C. (2022). Press release update. Colfax County Sheriff’s Office.

[gh270179-bib-0005] Barkjohn, K. K. , Gantt, B. , & Clements, A. L. (2021). Development and application of a united States‐wide correction for PM2.5 data collected with the PurpleAir sensor. Atmospheric Measurement Techniques, 14(6), 4617–4637. 10.5194/amt-14-4617-2021 PMC8422884 34504625

[gh270179-bib-0006] Barkjohn, K. K. , Holder, A. L. , Frederick, S. G. , & Clements, A. L. (2022). Correction and accuracy of PurpleAir PM2.5 measurements for extreme wildfire smoke. Sensors, 22(24), 9669. 10.3390/s22249669 36560038 PMC9784900

[gh270179-bib-0007] Bian, Q. , Ford, B. , Pierce, J. R. , & Kreidenweis, S. M. (2020). A decadal climatology of chemical, physical, and optical properties of ambient smoke in the Western and Southeastern United States. Journal of Geophysical Research: Atmospheres, 125(1), e2019JD031372. 10.1029/2019JD031372

[gh270179-bib-0008] Burke, M. , Childs, M. L. , De la Cuesta, B. , Qiu, M. , Li, J. , Gould, C. F. , et al. (2023). The contribution of wildfire to PM2.5 trends in the USA. Nature, 622(7984), 761–766. 10.1038/s41586-023-06522-6 37730996

[gh270179-bib-0009] Center for International Earth Science Information Network . (2023). Gridded population of the world, version 4.11 (GPWv4): Population count. NASA Socioeconomic Data and Applications Center (SEDAC), Revision 11. 10.7927/H4JW8BX5

[gh270179-bib-0010] Childs, M. L. , Li, J. , Wen, J. , Heft‐Neal, S. , Driscoll, A. , Wang, S. , et al. (2022). Daily local‐level estimates of ambient wildfire smoke PM2.5 for the contiguous US. Environmental Science & Technology, 56(19), 13607–13621. 10.1021/acs.est.2c02934 36134580

[gh270179-bib-0011] Delfino, R. J. , Brummel, S. , Wu, J. , Stern, H. , Ostro, B. , Lipsett, M. , et al. (2009). The relationship of respiratory and cardiovascular hospital admissions to the southern California wildfires of 2003. Occupational and Environmental Medicine, 66(3), 189–197. 10.1136/oem.2008.041376 19017694 PMC4176821

[gh270179-bib-0012] Ford, B. , Val Martin, M. , Zelasky, S. E. , Fischer, E. V. , Anenberg, S. C. , Heald, C. L. , & Pierce, J. R. (2018). Future fire impacts on smoke concentrations, visibility, and health in the contiguous United States. GeoHealth, 2(8), 229–247. 10.1029/2018GH000144 32159016 PMC7038896

[gh270179-bib-0013] Gan, R. W. , Ford, B. , Lassman, W. , Pfister, G. , Vaidyanathan, A. , Fischer, E. , et al. (2017). Comparison of wildfire smoke estimation methods and associations with cardiopulmonary‐related hospital admissions. GeoHealth, 1(3), 122–136. 10.1002/2017GH000073 28868515 PMC5580836

[gh270179-bib-0014] Gan, R. W. , Liu, J. , Ford, B. , O’Dell, K. , Vaidyanathan, A. , Wilson, A. , et al. (2020). The association between wildfire smoke exposure and asthma‐specific medical care utilization in Oregon during the 2013 wildfire season. Journal of Exposure Science and Environmental Epidemiology, 30(4), 618–628. 10.1038/s41370-020-0210-x 32051501 PMC8745685

[gh270179-bib-0015] Gasparrini, A. , Scheipl, F. , Armstrong, B. , & Kenward, M. G. (2017). A penalized framework for distributed lag non‐linear models. Biometrics, 73(3), 938–948. 10.1111/biom.12645 28134978

[gh270179-bib-0016] Global Asthma Network . (2022). The global asthma report. International Journal of Tuberculosis & Lung Disease, 26(1), 1–104. 10.5588/ijtld.22.1010 36303302

[gh270179-bib-0017] Hahn, M. B. , Kuiper, G. , O’Dell, K. , Fischer, E. V. , & Magzamen, S. (2021). Wildfire smoke is associated with an increased risk of cardiorespiratory emergency department visits in Alaska ‐ Hahn ‐ 2021 ‐ GeoHealth. Wiley Online Library. 10.1029/2020GH000349 PMC8137270 34036208

[gh270179-bib-0018] Hand, J. L. , Prenni, A. J. , Raffuse, S. M. , Hyslop, N. P. , Malm, W. C. , & Schichtel, B. A. (2024). Spatial and seasonal variability of remote and urban speciated fine particulate matter in the United States. Journal of Geophysical Research: Atmospheres, 129(23), e2024JD042579. 10.1029/2024JD042579 PMC11608814 39624178

[gh270179-bib-0019] Hazard Mapping System Smoke Product . (2022). National Oceanic and atmospheric administration [Dataset]. Retrieved from https://satepsanone.nesdis.noaa.gov/pub/FIRE/web/HMS/Smoke_Polygons/Shapefile/2022/

[gh270179-bib-0020] Hodshire, A. L. , Akherati, A. , Alvarado, M. J. , Brown‐Steiner, B. , Jathar, S. H. , Jimenez, J. L. , et al. (2019). Aging effects on biomass burning aerosol mass and composition: A critical review of field and laboratory studies. Environmental Science & Technology, 53(17), 10007–10022. 10.1021/acs.est.9b02588 31365241

[gh270179-bib-0021] Holstius, D. M. , Reid, C. E. , Jesdale, B. M. , & Morello, ‐F. R. (2012). Birth weight following pregnancy during the 2003 Southern California wildfires. Environmental Health Perspectives, 120(9), 1340–1345. 10.1289/ehp.1104515 22645279 PMC3440113

[gh270179-bib-0022] Hulburd, A. (2022). Suppression repair continues despite rains on the black fire. Retrieved from https://nmfireinfo.com/2022/06/22/suppression‐repair‐continues‐despite‐rains‐on‐the‐black‐fire/

[gh270179-bib-0023] InciWeb . (2022a). Incident information. Retrieved from http://inciweb.wildfire.gov/incident‐information/nmcif‐bear‐trap‐fire

[gh270179-bib-0024] InciWeb . (2022b). InciWeb. Retrieved from http://inciweb.wildfire.gov/incident‐information/nmsnf‐cerro‐pelado

[gh270179-bib-0025] InciWeb . (2024). Incident information. Retrieved from http://inciweb.wildfire.gov/incident‐information/nmn4s‐cooks‐peak

[gh270179-bib-0026] Isaaks, E. H. , & Srivastava, R. M. (1989). Applied geostatistics. Oxford University Press.

[gh270179-bib-0027] Jaffe, D. A. , Miller, C. , Thompson, K. , Finley, B. , Nelson, M. , Ouimette, J. , & Andrews, E. (2023). An evaluation of the U.S. EPA’s correction equation for PurpleAir sensor data in smoke, dust, and wintertime urban pollution events. Atmospheric Measurement Techniques, 16(5), 1311–1322. 10.5194/amt-16-1311-2023

[gh270179-bib-0028] Janes, H. , Sheppard, L. , & Lumley, T. (2005a). Case–crossover analyses of air pollution exposure data: Referent selection strategies and their implications for bias. Epidemiology, 16(6), 717–726. 10.1097/01.ede.0000181315.18836.9d 16222160

[gh270179-bib-0029] Janes, H. , Sheppard, L. , & Lumley, T. (2005b). Overlap bias in the case‐crossover design, with application to air pollution exposures. Statistics in Medicine, 24(2), 285–300. 10.1002/sim.1889 15546133

[gh270179-bib-0030] Janssen, S. , Dumont, G. , Fierens, F. , & Mensink, C. (2008). Spatial interpolation of air pollution measurements using CORINE land cover data. Atmospheric Environment, 42(20), 4884–4903. 10.1016/j.atmosenv.2008.02.043

[gh270179-bib-0031] Johnston, F. H. , Kavanagh, A. M. , Bowman, D. M. J. S. , & Scott, R. K. (2002). Exposure to bushfire smoke and asthma: An ecological study. Medical Journal of Australia, 176(11), 535–538. 10.5694/j.1326-5377.2002.tb04551.x 12064985

[gh270179-bib-0032] Kuula, J. , Mäkelä, T. , Aurela, M. , Teinilä, K. , Varjonen, S. , González, Ó. , & Timonen, H. (2020). Laboratory evaluation of particle‐size selectivity of optical low‐cost particulate matter sensors. Atmospheric Measurement Techniques, 13(5), 2413–2423. 10.5194/amt-13-2413-2020

[gh270179-bib-0033] Lassman, W. , Ford, B. , Gan, R. W. , Pfister, G. , Magzamen, S. , Fischer, E. V. , & Pierce, J. R. (2017). Spatial and temporal estimates of population exposure to wildfire smoke during the Washington state 2012 wildfire season using blended model, satellite, and in situ data. GeoHealth, 1(3), 106–121. 10.1002/2017GH000049 32158985 PMC7007107

[gh270179-bib-0034] Liu, J. C. , Pereira, G. , Uhl, S. A. , Bravo, M. A. , & Bell, M. L. (2015). A systematic review of the physical health impacts from non‐occupational exposure to wildfire smoke. Environmental Research, 136, 120–132. 10.1016/j.envres.2014.10.015 25460628 PMC4262561

[gh270179-bib-0035] Magzamen, S. , Gan, R. W. , Liu, J. , O’Dell, K. , Ford, B. , Berg, K. , et al. (2021). Differential cardiopulmonary health impacts of local and long‐range transport of wildfire smoke. GeoHealth, 5(3), e2020GH000330. 10.1029/2020GH000330 PMC8900982 35281479

[gh270179-bib-0036] Maji, K. J. , Ford, B. , Li, Z. , Hu, Y. , Hu, L. , Langer, C. E. , et al. (2024). Impact of the 2022 New Mexico, US wildfires on air quality and health. Science of The Total Environment, 946, 174197. 10.1016/j.scitotenv.2024.174197 38914336

[gh270179-bib-0037] Malings, C. , Tanzer, R. , Hauryliuk, A. , Saha, P. K. , Robinson, A. L. , Presto, A. A. , & Subramanian, R. (2020). Fine particle mass monitoring with low‐cost sensors: Corrections and long‐term performance evaluation. Aerosol Science and Technology, 54(2), 160–174. 10.1080/02786826.2019.1623863

[gh270179-bib-0038] Malm, W. C. , Sisler, J. F. , Huffman, D. , Eldred, R. A. , & Cahill, T. A. (1994). Spatial and seasonal trends in particle concentration and optical extinction in the United States. Journal of Geophysical Research, 99(D1), 1347–1370. 10.1029/93JD02916

[gh270179-bib-0039] Molina Rueda, E. , Carter, E. , L’Orange, C. , Quinn, C. , & Volckens, J. (2023). Size‐resolved field performance of low‐cost sensors for particulate matter air pollution. Environmental Science and Technology Letters, 10(3), 247–253. 10.1021/acs.estlett.3c00030 36938150 PMC10018765

[gh270179-bib-0040] National Interagency Fire Center . (2025). WFIGS interagency fire perimeters. Retrieved from https://www.arcgis.com/sharing/rest/content/items/70ff5accb0ce45c68554d8fdd90aac10/info/metadata/metadata.xml?format=default&output=html

[gh270179-bib-0041] O’Dell, K. , Bilsback, K. , Ford, B. , Martenies, S. E. , Magzamen, S. , Fischer, E. V. , & Pierce, J. R. (2021). Estimated mortality and morbidity attributable to smoke plumes in the United States: Not just a Western US problem. GeoHealth, 5(9), e2021GH000457. 10.1029/2021GH000457 PMC8420710 34504989

[gh270179-bib-0042] O’Dell, K. , Ford, B. , Burkhardt, J. , Magzamen, S. , Anenberg, S. C. , Bayham, J. , et al. (2022). Outside in: The relationship between indoor and outdoor particulate air quality during wildfire smoke events in western US cities. Environmental Research: Health, 1(1), 015003. 10.1088/2752-5309/ac7d69

[gh270179-bib-0043] O’Dell, K. , Ford, B. , Fischer, E. V. , & Pierce, J. R. (2019). Contribution of wildland‐fire smoke to US PM2.5 and its influence on recent trends. Environmental Science & Technology, 53(4), 1797–1804. 10.1021/acs.est.8b05430 30681842

[gh270179-bib-0044] Ouimette, J. R. , Malm, W. C. , Schichtel, B. A. , Sheridan, P. J. , Andrews, E. , Ogren, J. A. , & Arnott, W. P. (2022). Evaluating the PurpleAir monitor as an aerosol light scattering instrument. Atmospheric Measurement Techniques, 15(3), 655–676. 10.5194/amt-15-655-2022

[gh270179-bib-0045] Rappold, A. G. , Stone, S. L. , Cascio, W. E. , Neas, L. M. , Kilaru, V. J. , Carraway, M. S. , et al. (2011). Peat bog wildfire smoke exposure in rural North Carolina is associated with cardiopulmonary emergency department visits assessed through syndromic surveillance. Environmental Health Perspectives, 119(10), 1415–1420. 10.1289/ehp.1003206 21705297 PMC3230437

[gh270179-bib-0046] Reid, C. E. , Brauer, M. , Johnston, F. H. , Jerrett, M. , Balmes, J. R. , & Elliott, C. T. (2016). Critical review of health impacts of wildfire smoke exposure. Environmental Health Perspectives, 124(9), 1334–1343. 10.1289/ehp.1409277 27082891 PMC5010409

[gh270179-bib-0047] Riss, C. S. , Faulstich, S. D. , Reuther, P. S. , Metcalf, W. J. , Darrow, L. A. , Holmes, H. A. , & Strickland, M. J. (2025). Influence of fire characteristics on the associations between smoke PM2.5 exposure and acute cardiorespiratory health events. Environment International, 201, 109577. 10.1016/j.envint.2025.109577 40480103 PMC12208789

[gh270179-bib-0048] Sablan, O. (2025). New Mexico PM2.5 concentrations by ZIP code for 4 smoke exposure estimates (version 2) [Dataset]. Dryad. 10.5061/DRYAD.GB5MKKX2F

[gh270179-bib-0049] Sablan, O. , Ford, B. , Gargulinski, E. , Henery, G. L. , Nowell, H. , Rosen, Z. , et al. (2026). Characterizing particulate matter impacts of smoke from 2022 to 2023 agricultural burning in south Florida. GeoHealth, 10(1), e2025GH001365. 10.1029/2025GH001365 PMC12836378 41608314

[gh270179-bib-0050] Sayahi, T. , Butterfield, A. , & Kelly, K. E. (2019). Long‐term field evaluation of the plantower PMS low‐cost particulate matter sensors. Environmental Pollution, 245, 932–940. 10.1016/j.envpol.2018.11.065 30682749

[gh270179-bib-0051] Stowell, J. D. , Geng, G. , Saikawa, E. , Chang, H. H. , Fu, J. , Yang, C.‐E. , et al. (2019). Associations of wildfire smoke PM2.5 exposure with cardiorespiratory events in Colorado 2011–2014. Environment International, 133, 105151. 10.1016/j.envint.2019.105151 31520956 PMC8163094

[gh270179-bib-0052] Tryner, J. , L’Orange, C. , Mehaffy, J. , Miller‐Lionberg, D. , Hofstetter, J. C. , Wilson, A. , & Volckens, J. (2020). Laboratory evaluation of low‐cost PurpleAir PM monitors and in‐field correction using co‐located portable filter samplers. Atmospheric Environment, 220, 117067. 10.1016/j.atmosenv.2019.117067

[gh270179-bib-0053] Tunby, P. , Nichols, J. , Kaphle, A. , Khandelwal, A. S. , Van Horn, D. J. , & González‐Pinzón, R. (2023). Development of a general protocol for rapid response research on water quality disturbances and its application for monitoring the largest wildfire recorded in New Mexico, USA. Frontiers in Water, 5, 1223338. 10.3389/frwa.2023.1223338

[gh270179-bib-0054] US Census Bureau . (2010). TIGER/line shapefiles. Retrieved from https://www.census.gov/geographies/mapping‐files/time‐series/geo/tiger‐line‐file.html

[gh270179-bib-0055] US Census Bureau . (2023). American community survey 5‐Year data (2009‐2021). Retrieved from https://www.census.gov/data/developers/data‐sets/acs‐5year.html

[gh270179-bib-0056] U.S. Census Bureau . (2024). U.S. Census Bureau QuickFacts: New Mexico. Retrieved from https://www.census.gov/quickfacts/fact/table/NM/PST045223

[gh270179-bib-0057] US EPA, O. (2017). National emissions inventory (NEI) data. [Other Policies and Guidance] Retrieved from https://www.epa.gov/air‐emissions‐inventories/2017‐national‐emissions‐inventory‐nei‐data

[gh270179-bib-0058] US EPA, O. (2020). 2020 national emissions inventory (NEI) data. [Other Policies and Guidance] Retrieved from https://www.epa.gov/air‐emissions‐inventories/2020‐national‐emissions‐inventory‐nei‐data

[gh270179-bib-0059] Volckens, J. (2024). Summertime trends in U.S. population exposure to fine particulate air pollution (PM2.5) from wildfire smoke. Environmental Science and Technology Letters, 11(11), 1182–1186. 10.1021/acs.estlett.4c00770

[gh270179-bib-0060] Westerling, A. L. (2016). Increasing Western US forest wildfire activity: Sensitivity to changes in the timing of spring. Philosophical Transactions of the Royal Society B: Biological Sciences, 371(1696), 20150178. 10.1098/rstb.2015.0178 PMC4874415 27216510

