# Supplementary material for: Wildfire Smoke and Cardiorespiratory Emergency Visits in New Mexico 2022: Sensitivity to Exposure Estimates and Referent Periods
Source: Geohealth. 2026 Jul 14;10(7):e2025GH001492. doi: 10.1029/2025GH001492 (PMC13366114; doi:10.1029/2025GH001492)
Supplement: Supplementary file 1 — Supporting Information S1 [file GH2-10-e2025GH001492-s001.pdf]

GeoHealth

Supporting Information for

Wildfire smoke and cardiorespiratory emergency visits in New Mexico 2022: sensitivity to exposure estimates and referent periods

Olivia Sablan[1], Bonne Ford[2], Colin B. Hawkinson[3], Leiqiu Hu[4], Jihoon Jung[5], Chelsea Eastman Langer[3], Courtney Maichak[6], Kamal Jyoti Maji[7], Stephanie Moraga-McHaley[3], Armistead G. Russell[7], Christopher K. Uejio[8], Melissa VanSickle[8], Emily V. Fischer[1], Jeffrey R. Pierce[1], Sheryl Magzamen[6]

Colorado State University, Department of Atmospheric Science[1]

Colorado State University, Cooperative Institute for Research in the Atmosphere[2]

New Mexico Department of Health[3]

University of Alabama Huntsville, Department of Atmospheric and Earth Science[4]

University of North Texas, Department of Geography and the Environment[5]

Colorado State University, Department of Environmental and Radiological Health Sciences[6]

Georgia Institute of Technology, Department of Civil and Environmental Engineering[7]

Florida State University, Department of Geography[8]

Contents of this file

Figures S1 to S5
Tables S1 to S2

Introduction

This supplemental material provides additional context and details essential for a comprehensive understanding of various aspects related to our findings. It details results using different variogram ordinary kriging parameters (i.e., sill, range, nugget) for smoke product that uses measurements from the Environmental Protection Agency Air Quality System (EPA AQS) and low-cost PurpleAir sensors. We also provide a comparison of the four gridded smoke product estimates ($PM_{2.5}$ from the EPA regulatory-grade monitors; $PM_{2.5}$ from both the EPA regulatory-grade monitors and low-cost PurpleAir observations; modeled 24-hour average wildfire smoke $PM_{2.5}$ from the Community Multiscale Air Quality Modeling System (CMAQ); CMAQ daily 1-hour maximum wildfire smoke $PM_{2.5}$) to the corresponding in situ measurements. A table detailing the ICD-10-CM codes for each health outcome is also given in this document. Additionally, we provide a table to summarize cardiorespiratory-related Emergency Department (ED) visits in NM during April 6 – August 22, 2022, which was during the Calf Canyon/Hermit's Peak fire. To give context for the monitors most impacted by smoke, we showed the percentage of days with a NOAA Hazard Mapping System (HMS) smoke plume during the Calf Canyon/Hermit's Peak wildfire. Lastly, we provide results for all health outcomes using the two different referent periods and the two smoke exposure products the use in situ measurements. Overall, this supplement should be used to supplement understanding of the results and method used in the manuscript.

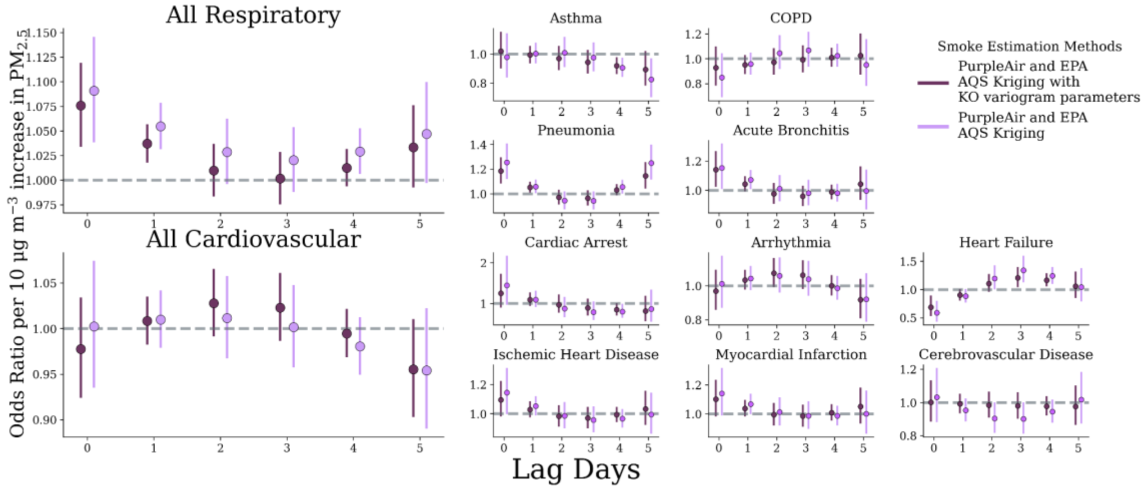

Figure S1. Distributed lag results for April 1 - September 30, 2022, ED visits and the Regulatory + PA smoke product with the same variogram parameters as the O'Dell et al. (2019) smoke product (light purple) and the Regulatory + PA smoke product with optimized variogram parameters (dark purple).

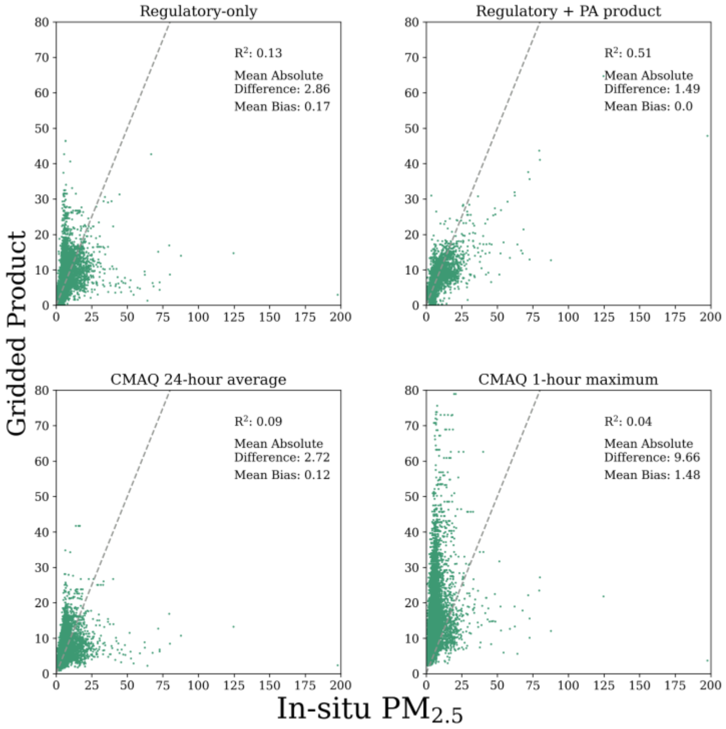

Figure S2. Comparison of 24-hour in situ measurements from PurpleAir and the regulatory monitors to the corresponding grid of total $PM_{2.5}$ product/model from April 6 - August 22, 2022. The Pearson correlation ("R^2"), mean absolute difference (μg m^{-3}), and mean bias (%) are displayed on the corresponding subplot. The gray dashed line is the 1-1 line.

Table S1. Emergency department ICD-10 codes per diagnosis

Diagnosis	ICD-10-CM codes
All Respiratory	J00-J98
Asthma	J45
COPD	J44
Pneumonia	J12-J18
Bronchitis	J20-J22
All cardiovascular	I00-I78
Cardiac arrest	I46
Arrhythmia	I47-I49
Heart failure	I50
Ischemic heart disease	I20-I22, I24-I25
Myocardial infarction	I21, I22
Cerebrovascular disease	I60-I163, I65-I69, G45, I23

Table S2. Summary of cardiorespiratory-related Emergency Department (ED) visits in New Mexico between April 6 - August 22, 2022. American Indian/Alaskan Native is abbreviated as AIAN, and Asian/Native Hawaiian/Pacific Islander is abbreviated as AANHPI. Corresponding ICD-10 codes are provided in Table S1.

		**Age Category**			**Sex**		**Race/Ethnicity**					
**Health Outcomes**	**Cases(n)**	**<15 (%)**	**15 to 65 (%)**	**>65 (%)**	**Male (%)**	**Female (%)**	**AIAN (%)**	**AANPHI (%)**	**Black (%)**	**Hispanic (%)**	**White (%)**	**Unknown (%)**
All respiratory	21,223	29.8	48.4	21.8	46.4	53.6	9.3	0.6	2.6	49.0	34.3	4.2
Asthma	2,160	29.3	59.7	11.0	43.2	56.8	8.4	0.6	4.5	52.9	29.4	4.1
COPD	1,383	0.2	33.6	66.2	44.8	55.2	1.4	0.5	2.2	29.7	62.6	3.5
Pneumonia	3,789	14.2	41.1	44.7	50.8	49.2	6.2	0.5	2.4	42.2	45.3	3.4
Acute bronchitis	2,799	44.6	40.9	14.5	46.6	53.4	8.8	0.2	2.1	55.6	30.0	3.4
All Cardiovascular	11,651	0.3	41.0	58.7	54.0	46.0	3.9	0.8	2.5	36.9	51.5	4.4
Cardiac Arrest	373	3.2	44.2	51.5	59.2	40.8	5.1	1.1	4.0	38.6	38.9	12.3
Arrhythmia	2,329	0.2	34.4	65.4	52.7	47.3	3.3	0.9	0.9	25.3	64.7	4.9
Heart Failure	656	–	38.9	61.1	55.0	45.0	4.0	0.9	1.8	37.2	52.6	3.5
Ischemic Heart Disease	2,689	–	43.9	56.1	36.4	38.7	2.8	0.9	2.1	40.6	49.8	3.9
Myocardial Infarction	2,380	–	44.4	55.6	64.1	35.9	2.6	1.0	2.1	41.3	49.0	4.0
Cerebrovascular Disease	2,395	0.3	32.8	66.8	51.0	49.0	5.4	1.0	2.0	37.4	50.5	3.7
**TOTAL**	**33,266**	**6,357**	**15,152**	**11,753**	**16,308**	**16,955**	**2,442**	**233**	**838**	**4,832**	**13,498**	**1,423**

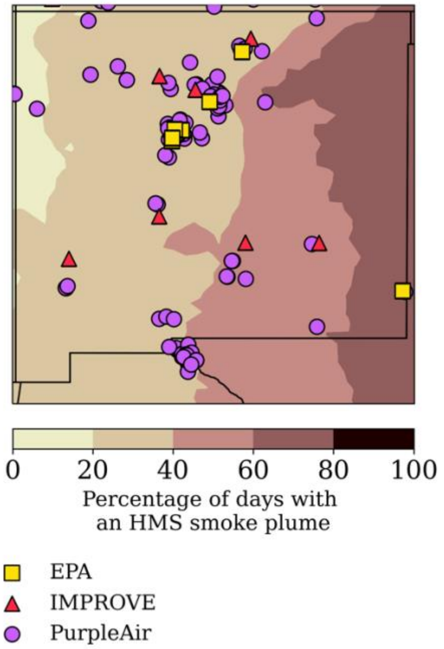

Figure S3. Percentage of days with a NOAA Hazard Mapping System (HMS) smoke plume during the Calf Canyon/Hermit's Peak wildfire (April 6 - August 22, 2022). Note: HMS is not a surface-based product; therefore, the smoke plumes used in this figure may be aloft and not contributing to ground $PM_{2.5}$ concentrations. Ground-based $PM_{2.5}$ sensors are maps, including the Environmental Protection Agency Air Quality System (EPA AQS) (yellow squares), the Interagency Monitoring of PROtected Visual Environments (IMPROVE) monitors (red triangles), and the PurpleAir sensors (purple circles).

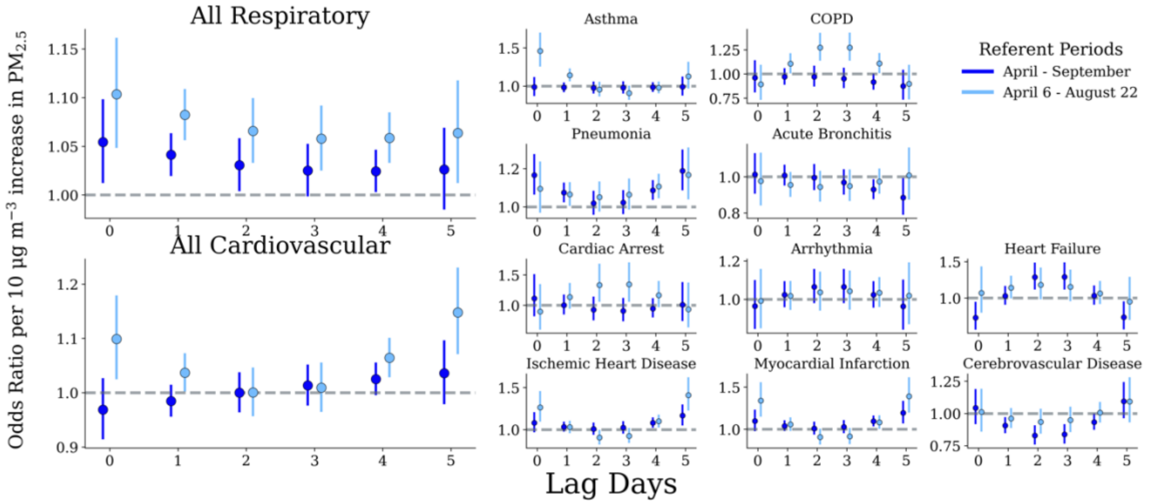

Figure S4. Distributed lag effects of a 10 µg m^{-3} increase in wildfire smoke $PM_{2.5}$ on likelihood of all cardiorespiratory-related emergency department visits during April - September 2022 (dark blue) and April 6 - August 22, 2022 (light blue) for the Regulatory-only smoke product.

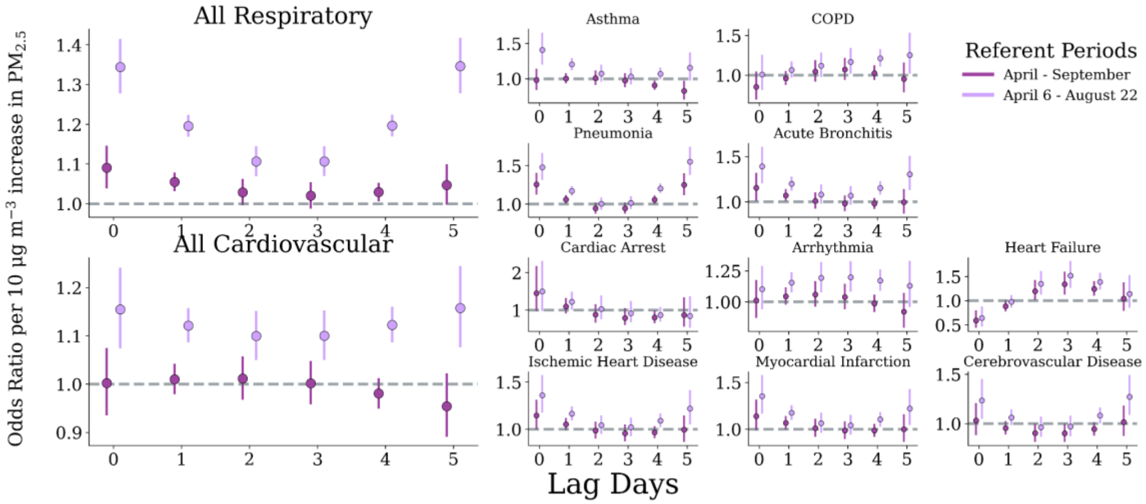

Figure S5. Distributed lag effects of a 10 μg m^{-3} increase in wildfire smoke $PM_{2.5}$ on likelihood of all cardiorespiratory-related emergency department visits during April - September 2022 (dark purple) and April 6 - August 22, 2022 (light purple) for the Regulatory + PA smoke product.
